# *Case of Uterine Hemorrhage, Occurring in a Patient of the* Middlesex Infirmary, *in Which Transfusion of Blood Was Employed Unsuccessfully*

**Published:** 1826-08

**Authors:** George Jewel


### UTERINE HEMORRHAGE.

*Case of Uterine Hemorrhage, occurring in a Patient of the* MIDDLESEX INFIRMARY, *in which Transfusion of Blood was employed unsuccessfully.* By GEORGE JEWEL, Esq.

ABOUT two o'clock on the morning of Friday, the 9th instant, a woman, of small stature and delicate constitution, who had previously been the subject of several very severe labours, was delivered, by the natural efforts, of a small dead child, after a difficult and protracted labour. There being some discharge of blood, the placenta was removed, without any obstacle, a few minutes after the birth of the child. Within half an hour the patient was seized with rigors, and complained of cold; and, upon examination, I found that a large quantity of blood had been effused, that rapid respiration and coldness of the extremities had supervened, and that the pulse was not perceptible at the wrist. Forty drops of Laudanum, and a drachm of Sp. Ammon. Aromat., were administered; and Mr. Allan, of Leicester-square, was sent for. Upon his arrival, the hemorrhage had ceased, at least externally; and the uterus, though not contracted into a hard tumor, did not appear to be distended. Pressure was made upon it by means of a band tightly applied round the body. Meanwhile the ghastly and sunken appearance of the patient's countenance,—her inability to remain a moment in any posture,—her pushing off the bedclothes, desiring to be raised up, tossing about her arms, and rapid breathing, seemed to threaten immediate dissolution. Eighty drops of laudanum, with a table-spoonful of brandy, were given in a little gruel, with the view of tranquillising the patient, and of inducing a disposition to slumber; which, however, was interrupted, in the course of five or ten minutes, by a return of the irresistible desire to change her position, tossing of the arms, and other alarming symptoms. The laudanum was, therefore, repeated in smaller doses, with the addition of a little brandy and gruel, every five or ten minutes, as the symptoms demanded.

As it was possible that hemorrhage might be going on internally, an examination was instituted by introducing the hand; but only two inconsiderable clots of blood were found within the uterus. These were removed, and a pillow, rolled firmly up, being applied over the belly, was bound tightly in that situation by a broad band round the waist.

The attempts to support the patient by the frequent repetition of laudanum and brandy, with the addition of carbonate of ammonia, having been continued for four or five hours, without improving the patient's condition; and the pulse being scarcely perceptible at the wrist, while the extremities had become cold, and the whole surface bedewed with a cold clammy moisture, the operation of transfusion suggested itself, as still affording a chance of saving the patient's life. Mr. Boyle, the other surgeon of the Infirmary, was sent for, and, he concurring in the propriety of the operation, it was performed with as little delay as circumstances would admit.

No vein could be found at the bend of the arm large enough to admit the small ivory tube of the syringe with which we were provided; and therefore the right external jugular vein was opened, and the tube being inserted, the syringe (which contained three drachms) was filled with blood drawn from the arm of the patient's husband into a small bason, which was placed within a larger one containing warm water; and due care being taken to expel every particle of air from the syringe, the blood was very gradually and gently injected through the tube into the jugular vein. The syringe was filled and discharged sixteen times in the space of about twenty minutes; but as, in turning up the point of the instrument, to expel any air which it might contain, some blood escaped each time, it was calculated that little more than four ounces were actually thrown into the vein. During the operation, the patient complained of being sick, and, towards the conclusion of it, the desire to change her posture became irresistible, and, by her turning her neck, the tube was displaced. With the exception of sickness, not the slightest deviation in the previous train of symptoms was produced.

There was now every indication of approaching dissolution, and, within a quarter of an hour from the termination of the operation, after several long sobs, she expired.

Permission was not obtained to examine the body until Monday afternoon, (the 12th.) Although the greatest care had been taken to prevent air from entering the vein during the operation, yet the possibility of some having been drawn in, (during the momentary removal of the finger from the end of the tube, to allow the point of the syringe to be inserted into it,) and thus perhaps contributed to accelerate the death of the patient, having been suggested, attention was especially directed to ascertain the fact. With this view, the superior and inferior vena cava, and the pulmonary artery, were secured with ligatures; the heart was then removed from its situation, and immersed in a bason of water. A tumbler, filled with water, being held inverted over the heart, the latter was punctured, and, on being pressed with the hand, a few minute bubbles of air escaped into the tumbler,—in quantity not amounting to a drachm. The heart contained very little coagulated blood. The uterus was quite empty. The antero-posterior diameter of the pelvis was barely three inches and a half, and the other dimensions were proportionally small. The promontory of the sacrum formed a sharp angular projection.

It may be a question whether the small quantity of air found in the right ventricle of the heart, was the product of putrefaction, or the remains of a larger portion of injected atmospheric air, the rest of which had been carried into the circulation?—Without pretending to determine this, and the numerous other interesting inquiries which the case suggests, I deem it a duty to submit this succinct statement to the consideration of the profession, as it can only be by an unreserved communication of such facts that a just estimate can be formed of the value of the operation of transfusion, as a means of saving life under circumstances where other measures have proved unavailing. The operation itself appears a reasonable one: at the same time, I cannot help expressing considerable doubt as to the propriety of anticipating any decidedly favourable result from the introduction of a few ounces of blood into the system of a patient sinking from a loss of several pounds. This doubt, however, is to be determined by general experience, and certainly not by a few successful or unsuccessful cases.

24, *Sackville-street, Piccadilly ; June* 13*th*, 1826.

---

### OVARIAN TUMOR.

*Case in which an Attempt was made to Extirpate Ovarian Tumors.*

By Dr. GRANVILLE.

